# Emerging Nano Bioinks in Bioprinting: Functional Materials, Engineering Strategies, and Biomedical Applications

**DOI:** 10.3390/ma19142957

**Published:** 2026-07-09

**Authors:** Adam Mohammed, Hailey Gibbons, Thais Muratori Holanda, Nicole Salazar, Eric Saliim, Darlene K. Taylor, Ufana Riaz

**Affiliations:** 1 Julius L. Chambers Biomedical/Biotechnology Research Institute (BBRI), North Carolina Central University, 1801 Fayetteville St., Durham, NC 27707, USA; 2 Department of Biological and Biomedical Sciences, Julius L. Chambers Biomedical/Biotechnology Research Institute (BBRI), North Carolina Central University, 1801 Fayetteville St., Durham, NC 27707, USA

**Keywords:** nano bioinks, fabrication, regenerative medicine, biosensing, drug delivery, tissue engineering

## Abstract

Nano bioinks have recently emerged as a promising class of biomaterials for advanced bioprinting applications, offering new opportunities in regenerative medicine, controlled drug delivery, and biosensing technologies. These materials are typically developed by integrating nanostructures such as nanoparticles, nanosheets, and nanofibers into polymeric or hydrogel matrices to enhance mechanical strength, bioactivity, and printing performance. Various fabrication approaches such as direct blending, in-situ polymerization, and surface functionalization are used to incorporate nanomaterials into bioink formulations. Subsequent crosslinking strategies are employed to improve print fidelity and structural stability while maintaining cell viability and biological functionality during the bioprinting process. Despite significant progress in recent years, several challenges continue to hinder the clinical translation of nano bioinks. Achieving consistent batch-to-batch reproducibility, ensuring long-term biocompatibility, and optimizing rheological properties for reliable printing remain critical issues. In addition, regulatory pathways and ethical considerations related to the biomedical use of nano-enabled bioinks are still insufficiently addressed in the literature. This review provides a comprehensive overview of recent advances in the design and fabrication of nano bioinks, highlighting key synthesis strategies, functional nanomaterials used in bioink formulations, and their emerging applications in tissue engineering, drug delivery, and biosensing. Furthermore, the review discusses the major technical, regulatory, and translational challenges that need to be addressed to facilitate the safe and effective implementation of nano bioinks in future biomedical applications.

## 1. Introduction

Three-dimensional (3D) bioprinting has rapidly evolved into a powerful interdisciplinary technology that integrates principles from materials science, tissue engineering, biomedical engineering, and regenerative medicine [1]. This technology enables the fabrication of highly organized biological constructs by depositing living cells, biomaterials, and bioactive molecules in a controlled and spatially defined manner. Unlike traditional scaffold fabrication techniques, 3D bioprinting allows precise control over cell placement, architecture, and microenvironment, making it possible to generate tissue models that more closely resemble native biological structures [2,3]. As a result, bioprinting has gained considerable attention for applications in tissue regeneration, disease modeling, drug screening, and personalized medicine. The global bioink market has experienced rapid expansion due to increasing research investment in tissue engineering and biomedical manufacturing. Recent market analysts estimate that the global bioink market is currently valued at approximately USD 2.58 billion and is projected to reach nearly USD 8.42 billion by 2034, corresponding to a compound annual growth rate (CAGR) of approximately 12.5% [4,5]. This growth reflects the increasing demand for advanced biomaterials capable of supporting the complex requirements of bioprinting technologies.

At the center of the bioprinting process lies the bioink, which serves as the functional material deposited by bioprinters to form biological structures. Bioinks are typically composed of biocompatible polymers, hydrogels, or composite materials that provide a supportive microenvironment for cells while maintaining the rheological properties required for printing [6]. An effective bioink must simultaneously satisfy several criteria, including appropriate viscosity, shear-thinning behavior, structural stability after printing, and the ability to maintain high cellular viability. In addition, bioinks should support cell adhesion [7], proliferation [8], differentiation [9], and extracellular matrix formation [10] after printing. Among the various classes of bioinks currently under investigation, nano bioinks have emerged as an advanced category of materials that incorporate nanoscale components into hydrogel/polymeric matrices. These nanoscale additives include carbon nanotubes [11], graphene oxide, MXene nanosheets, Au nanoparticles [12,13,14,15], and other engineered nanomaterials [14]. The integration of nanomaterials into bioink formulations significantly enhances their functional properties, including mechanical strength, electrical conductivity, bioactivity, and printability. Due to their extremely high surface-area-to-volume ratio and tunable surface chemistry, nanomaterials can interact efficiently with both polymer networks and biological cells, enabling the development of multifunctional bioink systems [15,16,17].

Bioink formulations are commonly derived from natural polymers, such as alginate [18], gelatin [19], collagen [20], fibrin [21], and hyaluronic acid [22], because of their inherent biocompatibility and similarity to components of the natural extracellular matrix. However, natural polymers alone often exhibit limited mechanical strength or rapid degradation. To address these limitations, researchers frequently combine natural polymers with synthetic materials or reinforcing agents to achieve improved structural stability, mechanical properties, and long-term functionality. Hybrid formulations that integrate both natural and synthetic components provide a balance between biological performance and mechanical durability, which is essential for maintaining structural integrity during and after the printing process [23].

Despite significant progress in bioink development, a fundamental challenge persists in balancing print fidelity and biological performance. Materials with high mechanical stiffness typically provide excellent shape retention and structural stability but may restrict cell spreading, migration, and proliferation. Conversely, softer hydrogels that promote cellular activity often lack the mechanical strength necessary to maintain structural integrity during printing. This inherent trade-off represents one of the primary obstacles in designing optimal bioink formulations [24,25].

Conventional hydrogel bioinks exhibit a trade-off between printability and biological performance. Increasing polymer concentration improves viscosity and printing fidelity but simultaneously reduces pore size, nutrient diffusion, cell migration, and cellular proliferation. Conversely, decreasing polymer concentration improves cytocompatibility but compromises structural stability after printing. The conventional bioinks generally possess poor mechanical strength, making it difficult to fabricate large or load-bearing tissues. Printed constructs often collapse under their own weight before complete crosslinking. Also, limited shear-thinning behavior and slow recovery after extrusion reduce printing precision and shape fidelity, particularly when printing complex overhanging structures. Furthermore, dense hydrogel networks restrict oxygen and nutrient transport, resulting in poor cell viability within thick constructs due to insufficient vascularization.

**Figure 1:**
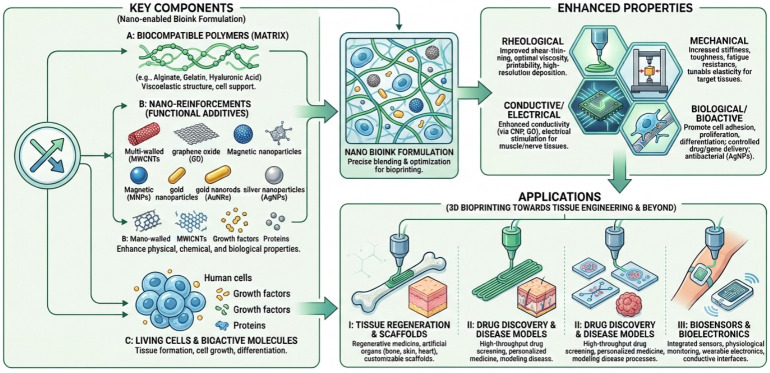
Overview of nano bioink formulation. The figure was created with assistance from Google Gemini and subsequently reviewed, edited, and scientifically validated by the authors.

The integration of nanomaterials into bioink systems has emerged as an effective strategy to address the intrinsic limitations of conventional hydrogel-based formulations. Nanomaterials enhance the mechanical, chemical, and biological performance of bioinks while maintaining cellular compatibility, as shown in Figure 1 [26,27]. Their unique physicochemical attributes, including high surface area, tunable functionality, and nanoscale dimensions, enable efficient interaction with polymer networks, resulting in improved structural integrity and bioactivity. Beyond mechanical reinforcement, nanomaterials introduce functional versatility to bioinks. For instance, nano silicates incorporated into gelatin methacrylate (GelMA) matrices have been shown to promote osteogenic differentiation of human mesenchymal stem cells without the need for exogenous growth factors [28]. Similarly, carbon-based nanomaterials such as graphene oxide and carbon nanotubes enhance electrical conductivity, which is critical for engineering electrically responsive tissues, including cardiac and neural constructs [29]. Importantly, nano bioinks enable the integration of advanced functionalities such as controlled drug release, enhanced cell–material interactions, and responsiveness to external stimuli. These properties significantly broaden their applicability in regenerative medicine, implantable devices, biosensing platforms, and in vitro disease models.

## 2. Functional Nanomaterials in Bioinks

The functional role of nanomaterials extends beyond reinforcement. Many nanomaterials enable controlled drug delivery, acting as reservoirs for therapeutic agents that can be released in a sustained or stimuli-responsive manner. Others influence cell–material interactions by modifying surface properties, thereby affecting cell adhesion, proliferation, and differentiation [30]. Furthermore, certain nanomaterials impart responsive behavior, allowing bioinks to react to environmental triggers such as pH, temperature, light, or electrical signals.

### 2.1. Nano Silicates

Nano silicate is widely used as a reinforcing agent in bioink formulations, Table 1. These materials exhibit a high aspect ratio and charged surfaces, enabling strong interactions with polymer chains. As a result, nanoclays significantly improve the rheological properties of bioinks, including shear-thinning behavior and viscosity recovery, which are critical for extrusion-based bioprinting. In addition to mechanical reinforcement, nano clays contribute to enhanced structural stability and shape fidelity of printed constructs. Their ability to form physically crosslinked networks within hydrogels improves resistance to deformation and collapse. Furthermore, nano clays can act as carriers for bioactive molecules, enabling controlled release and promoting cell proliferation and differentiation.

**Figure 2:**
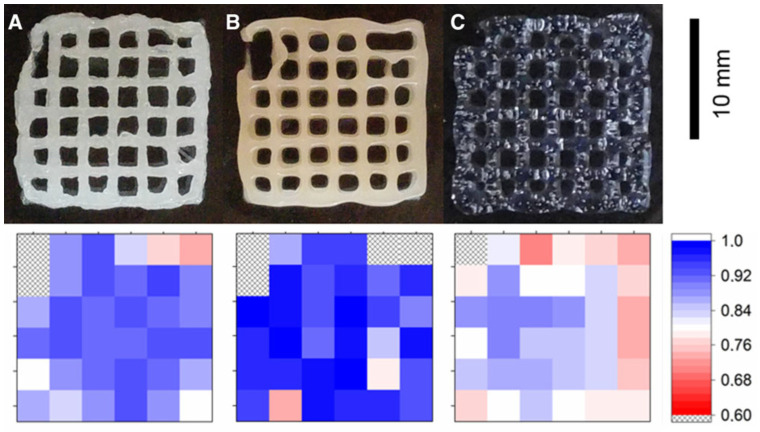
Quality control of the printed lattice structures. The upper row shows top-view images of the printed hydrogels using the additives DXG (**A**), BMA (**B**), and LRD (**C**). The bottom row shows the shape fidelity of the comparison with the desired structure, graded with a color code. Blue shades depict a high accuracy of printing and red a low conformity. Areas with printing errors such as hydrogel-filled pores and broken connections are marked with an “x.” Reproduced with permission from [31], Wiley, 2018.

Schmieg and co-workers [31] carried out a comparative study of the rheological and printing properties of a naturally purified hectorite (HT), marketed as Bentone^®^ MA, and Laponite^®^ RD (LAP) to optimize 3D bioprinting of an enzyme-loaded poly(ethylene glycol) diacrylate (PEGDA) bioink, without incorporating cells into the formulation, Figure 2.

Their results showed that HT exhibited higher viscosity than LAP but demonstrated weaker shear-thinning behavior. Beesetty et al. [32] studied montmorillonite (MMT) incorporated into a relatively low-cost, widely used high-density polyethylene (HDPE) matrix to develop filaments suitable for 3D printing applications. The HDPE composites were prepared with varying MMT loadings (0.5, 1, 2, and 5 wt.%) and evaluated through melt flow index (MFI) testing, which showed a progressive decrease in MFI with increasing MMT content, indicating reduced melt flowability. Kim et al. [33] fabricated polyethylene terephthalate glycol (PETG)–sepiolite composite via fused deposition modeling (FDM) that exhibited pronounced synergistic mechanical enhancement under tensile loading when compared with its injection-molded counterpart. Incorporation of 3 phr sepiolite led to a 35.4% increase in tensile strength for the 3D-printed PETG specimens, whereas only a 7.2% improvement was observed in injection-molded samples. Liu et al. [34] fabricated three-dimensional (3D) printed polymer scaffolds capable of generating tissue constructs with complex, hierarchical architectures and tunable mechanical and biological characteristics via polylactic acid (PLA) patterns modified with a polydopamine (PDA) interlayer to enable the stable immobilization of halloysite nanotubes (HNTs), thereby promoting guided cell alignment. In vitro studies with human mesenchymal stem cells (hMSCs) demonstrated that scaffold geometry played a critical role in directing cell orientation, with narrower stripe widths yielding more pronounced alignment; optimal performance was observed at a layer height of 0.05 mm. The HNT-functionalized PLA scaffolds exhibited improved cell adhesion and proliferation compared to unmodified counterparts. Treshkov et al. [35] designed a PA12/bentonite/kaolinite composite membrane via selective laser sintering (SLS), demonstrating the feasibility of producing polymer–mineral membranes using 3D printing techniques. The resulting membrane exhibited adequate flexural strength (22–29 MPa) and a modest yet consistent water permeability (~40 cm^3^/min). The successful fabrication further demonstrated the good flowability and uniform dispersion of the powder mixture, with no observable defects during laser sintering. Furka et al. [36] modified saponite with a cationic surfactant and subsequently functionalized it with the antimicrobial dye phloxine B (PhB). The stability and level of cytotoxicity of these materials in various physiological environments simulating the human body were studied. To preserve antimicrobial performance during printing, the optimal conditions were identified as 215 °C at 50 mm/s for polyethylene terephthalate-glycol (PETG) and SapPhB and 230 °C at 40 mm/s for PLA/SapPhB. Air filters fabricated via 3D printing from these materials retained 63.5% and 76.8% of their initial airflow for PLA/SapPhB and PETG/SapPhB, respectively. In contrast, analogous filters produced without PhB exhibited a complete loss of airflow.

### 2.2. Bioinks Based on Ag Nanoparticles (NPs)

Recent advances in 3D bioprinting have demonstrated the significant potential of Ag nanoparticles (NPs) incorporated into bioinks for the fabrication of multifunctional antimicrobial tissue engineering scaffolds, Table 2.

**Figure 3:**
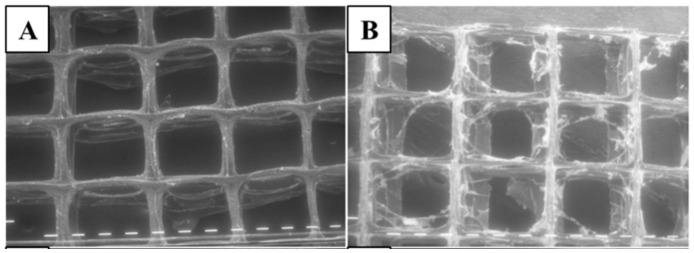
SEM pictures of 3D hydrogels. Calcium alginate hydrogel (**A**) without SSD and (**B**) containing SSD (magnification 40×). Reproduced with permission from [37], MDPI, 2020, under CC by 4.0 license.

Various studies have shown that integrating Ag NPs into hydrogel-based polymer systems enhances not only antibacterial performance but also the mechanical and functional characteristics of printed constructs. Bergonzi and co-workers’ [37] designed three-dimensional chitosan (CH)/alginate (ALG)-based hydrogels as multifunctional wound dressings for skin tissue regeneration. The alginate phase was loaded with 0.75% (*w*/*v*) silver sulfadiazine (SSD), a broad-spectrum antimicrobial agent routinely employed in the management of infected burn wounds. Four different CH/ALG hydrogel architectures were designed to investigate the influence of scaffold geometry on the controlled release kinetics of SSD while maintaining structural integrity and therapeutic efficacy, as shown in Figure 3.

Their findings revealed substantial antibacterial activity against both *Staphylococcus aureus* and *Pseudomonas aeruginosa*, while the addition of nanocrystalline cellulose improved scaffold stability and structural integrity during printing and post-fabrication handling. Similarly, a comprehensive review by Chakraborty et al. [38] highlighted the growing application of Ag NP-loaded hydrogel bioinks in bioprinting systems designed for tissue engineering and regenerative medicine. The review emphasized that extracellular matrix (ECM)-derived and synthetic hydrogel systems benefit considerably from silver nanoparticle incorporation because of their broad-spectrum antimicrobial behavior and enhanced functional bioactivity. The authors also noted that careful optimization of nanoparticle dispersion and concentration is essential to minimize cytotoxic effects and maintain cellular compatibility within bioprinted tissues. Additional evidence from regenerative bioprinting studies demonstrates that Ag NPs can provide immunomodulatory and infection-resistant properties in engineered tissues. Fang et al. [39] reported that Ag NPs containing hydrogel bioinks contribute to improved resistance against bacterial colonization and biofilm formation, making them promising candidates for wound healing and implantable biomedical constructs, thereby supporting tissue regeneration under infected or compromised healing conditions. Several investigations involving alginate-based composite scaffolds have also confirmed the effectiveness of Ag incorporation for antimicrobial wound dressing applications [40]. In these systems, low concentrations of silver nanoparticles were sufficient to inhibit both Gram-positive and Gram-negative bacterial strains, demonstrating minimum inhibitory concentration (MIC) values in the range of approximately 10 µg/mL. These findings suggest that carefully controlled nanoparticle loading can achieve potent antibacterial functionality while reducing the risk of excessive cytotoxicity. Studies involving agarose/Ag NPs composite printing demonstrated improved optical behavior, nanoscale responsiveness, and enhanced functional properties suitable for biosensing applications [40]. These multifunctional characteristics indicate that Ag NP loaded bioinks support the development of intelligent tissue scaffolds capable of combining regenerative, antimicrobial, and diagnostic functions within a single biofabricated platform. While challenges related to nanoparticle dispersion, long-term safety, and regulatory translation remain, current research strongly supports the potential of Ag NP-based bioinks for producing mechanically stable, antimicrobial, and multifunctional tissue engineering constructs for future biomedical use.

### 2.3. Zinc Oxide Nanoparticles-Based Bioinks

Zinc oxide nanoparticles (ZnO NPs) have gained significant attention in 3D bioprinting and tissue engineering because of their multifunctional properties, including antibacterial activity, mechanical reinforcement, thermal stability, osteogenic potential, and biocompatibility, Table 3. The incorporation of ZnO NPs into polymeric bioinks and printed scaffolds has demonstrated considerable promise for improving the structural and biological performance of biomedical constructs intended for regenerative medicine, wound healing, biosensing, and bone tissue engineering applications.

**Figure 4:**
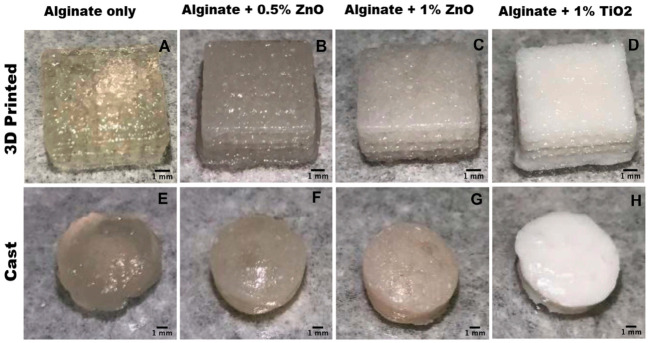
(**A**–**D**) depict 3D printed lattice structures. (**E**–**H**) portrays manually cast structures. Scale bar in all images depicts 1 mm. Reproduced with permission from [37], Dove Press, 2020, under CC by 4.0 license.

Cleetus et al. [41] investigated alginate-based hydrogels reinforced with low concentrations of ZnO NPs and reported significant improvements in mechanical stiffness and antibacterial activity, as shown in Figure 4.

The study demonstrated that ZnO NPs ranging from approximately 0.5% to 1% provided effective inhibition against bacterial growth while preserving favorable cytocompatibility. Importantly, the reinforced hydrogels maintained high levels of cell survival, indicating the suitability of ZnO NPs incorporated bioinks for tissue engineering applications where both structural integrity and biological compatibility are required. In thermoplastic polymer printing systems, ZnO NPs have also been explored as reinforcing agents for fused deposition modeling (FDM)-based fabrication. Kumar and colleagues [42] studied polylactic acid (PLA)/ZnO nanocomposites and observed that ZnO incorporation improved polymer crystallinity, thermal resistance, and overall material stability. These enhancements contributed to the development of mechanically robust printed structures with potential applications in biomedical devices and biosensor fabrication. The improved thermal behavior and functional properties of PLA/ZnO composites suggested their usefulness for producing durable and responsive 3D printed platforms.

More recent investigations involving gelatin/ZnO hydrogel systems have further highlighted the multifunctional advantages of ZnO NP incorporation in extrusion bioprinting [43]. Gelatin-based bioinks reinforced with ZnO NPs demonstrated substantial improvements in tensile strength, with some studies reporting increases of up to 67% compared to non-reinforced hydrogels. In addition to enhanced mechanical performance, these hybrid scaffolds exhibited strong antibacterial behavior against pathogenic microorganisms while maintaining cell viability levels above 85%, indicating that ZnO nanoparticles can simultaneously improve structural reinforcement and biological functionality. ZnO NP have also shown significant promise in bone tissue engineering applications through their osteogenic and antibacterial capabilities. In ZnO-modified ceramic scaffold systems composed of barium titanate (BT) and hydroxyapatite (HA), the incorporation of ZnO NPs provided effective antimicrobial properties while supporting bone regeneration under external stimulation conditions [44]. These findings indicate that ZnO NPs composite scaffolds may be particularly useful for orthopedic implants and bone repair applications where infection prevention and tissue integration are critical. Nanomaterial-enhanced bioinks have consistently emphasized the advantages of ZnO nanoparticles due to their relatively low toxicity, favorable biocompatibility, and broad-spectrum antimicrobial behavior [45]. Compared with other metallic nanoparticles, ZnO NPs exhibit strong biomedical compatibility while also contributing to enhanced scaffold stability, bioactivity, and cellular interactions. These characteristics make ZnO NPs attractive candidates for multifunctional hydrogel systems designed for regenerative medicine and advanced biofabrication technologies. The integration of ZnO NPs into bioinks and 3D-printed biomaterials has been widely investigated for multifunctional scaffolds with enhanced antibacterial performance, mechanical stability, and regenerative potential. Continued advances in nanomaterial engineering, smart hydrogel design, and biofabrication technologies are expected to further expand the biomedical applications of ZnO-incorporated bioprinted systems.

### 2.4. Graphene and Carbon Nanotubes

Carbon-based nanomaterials have become increasingly important in the development of advanced bioinks and multifunctional scaffolds for 3D bioprinting applications, Table 4. Among the most widely investigated carbon nanostructures are graphene-based materials, carbon nanotubes (CNTs), and carbon nanodots (CNDs/CDs) [46].

**Figure 5:**
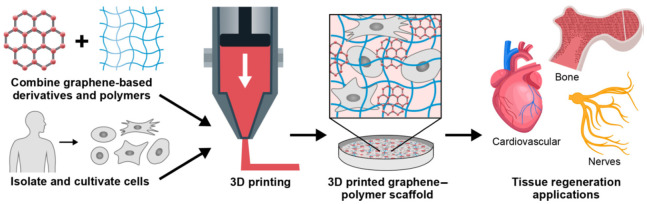
Schematic of generating 3D-printed tissue-engineered graphene-based scaffolds. Graphene-derived materials combined with cells are 3D printed to generate scaffolds for bone, neural, and cardiac tissue regeneration. Reproduced with permission from [47], MDPI, 2024, under CC by 4.0 license.

These nanomaterials possess distinct physicochemical, electrical, mechanical, and biological properties that influence their suitability for tissue engineering, biosensing, drug delivery, and regenerative medicine. Their incorporation into hydrogel bioinks and polymer composites has enabled the fabrication of highly functional bioprinted constructs with improved structural stability, conductivity, and biological responsiveness. Graphene-based systems, including graphene oxide (GO) and reduced graphene oxide (rGO), are among the most extensively studied nanomaterials in biofabrication due to their exceptional mechanical strength, electrical conductivity, and surface functionality [47], Figure 5.

The oxygen-containing functional groups present in graphene oxide improve its dispersibility in aqueous hydrogel systems such as gelatin methacrylate (GelMA), polyethylene glycol diacrylate (PEGDA), polyurethane, and alginate-based bioinks [46]. These surface functionalities also enhance rheological performance by promoting shear-thinning behavior, which is highly beneficial for extrusion-based bioprinting processes. Graphene-reinforced bioinks have been shown to significantly improve scaffold stiffness, Young’s modulus, and overall structural integrity while maintaining print fidelity. The rGO provides high electrical conductivity, making it highly suitable for engineering electroactive tissues such as neural, cardiac, and muscle tissues. Additionally, graphene-based scaffolds have demonstrated the ability to promote stem cell differentiation, including neural and chondrogenic lineage development, through enhanced cellular interactions and bioactive signaling. Despite these advantages, excessive graphene loading may lead to nanoparticle aggregation, oxidative stress, and dose-dependent cytotoxicity, which remain important considerations for biomedical translation. Carbon nanotubes, including single-walled carbon nanotubes (SWCNTs) and multi-walled carbon nanotubes (MWCNTs), have also attracted significant attention because of their outstanding tensile strength, electrical conductivity, and high aspect ratio. CNTs are commonly incorporated into polymer matrices such as polylactic acid (PLA), polycaprolactone (PCL), and hydrogel composites to produce mechanically reinforced and electrically conductive scaffolds. Their superior conductivity makes them particularly valuable for biosensors, bioelectronic interfaces, and tissue engineering applications requiring electrical stimulation. CNT-based scaffolds can effectively support signal transmission in neural and cardiac tissues while simultaneously improving the mechanical durability of printed constructs. However, one of the major limitations associated with CNTs is their poor dispersibility within aqueous bioink systems. Surface functionalization, chemical modification, or the use of surfactants is often required to achieve uniform nanoparticle distribution and stable rheological behavior. Furthermore, CNT biocompatibility remains highly dependent on particle purity, concentration, size, and surface chemistry, as improperly dispersed or highly concentrated CNTs may induce inflammatory responses and cytotoxicity. Carbon nanodots (CNDs), also referred to as carbon dots (CDs), represent a newer class of nanoscale carbon materials characterized by their ultrasmall size, fluorescence properties, and high biocompatibility. Unlike graphene and CNTs, carbon nanodots are primarily utilized for bioimaging, fluorescence tracking, biosensing, and controlled drug delivery applications rather than structural reinforcement. Due to their small dimensions and hydrophilic surface chemistry, carbon dots disperse efficiently within hydrogel matrices such as gelatin, chitosan, and alginate-based bioinks without significantly altering viscosity or printability. Their excellent cellular uptake, low toxicity, and photoluminescent behavior make them highly promising for theranostic applications involving simultaneous tissue regeneration and real-time imaging. Although carbon nanodots exhibit moderate electrical conductivity and mild antibacterial activity associated with reactive oxygen species (ROS) generation, their mechanical reinforcement capability is generally lower than that of graphene and CNT systems. Graphene-based materials are particularly valuable for enhancing mechanical performance, conductivity, and stem cell differentiation in electroactive tissue engineering applications [47,48]. Carbon nanotubes provide exceptional conductive reinforcement and are widely explored for biosensing and electrically responsive scaffolds. In contrast, carbon nanodots offer outstanding fluorescence properties, biocompatibility, and imaging functionality suitable for smart biomedical systems and targeted therapeutic delivery [49]. Aggregation of graphene and CNTs can negatively affect cellular interactions and scaffold homogeneity, while potential nanotoxicity remains an important area of investigation.

## 3. Performance Layer: Functional Outcomes of Nano Bioinks

The performance layer of nano bioinks refers to the functional outcomes achieved after incorporating nanomaterials into bioink systems for 3D bioprinting applications. Nano bioinks significantly enhance mechanical strength, electrical conductivity, print fidelity, and biological activity, enabling the fabrication of structurally stable and physiologically relevant tissue constructs. Depending on the type of nanomaterial used, these systems can promote stem cell differentiation, improve cellular adhesion and proliferation, support vascularization, and provide antimicrobial protection. Conductive nanomaterials such as graphene and carbon nanotubes facilitate electrical signaling in neural and cardiac tissues, while metallic and ceramic nanoparticles contribute to osteogenic activity, wound healing, and infection resistance. Additionally, fluorescent carbon dots and multifunctional nanoparticles enable biosensing, imaging, and controlled drug delivery within bioprinted scaffolds. The performance layer determines how effectively nano bioinks translate material design into functional biological and therapeutic outcomes for regenerative medicine and biomedical engineering applications.

### 3.1. Print Fidelity and Structural Stability

Print fidelity and structural stability in nanomaterial-reinforced 3D printing inks are governed primarily by rheological behavior, particularly viscosity, yield stress, and shear-thinning characteristics. Among the materials considered, silicate-based additives such as nano silicates (e.g., Laponite) consistently demonstrate superior performance due to their ability to form reversible, physically crosslinked networks that impart strong shear-thinning and rapid post-extrusion recovery, thereby enabling excellent shape retention and layer stacking [50,51]. In contrast, carbon-based nanomaterials exhibit more variable behavior: graphene oxide (GO) enhances print fidelity by improving dispersion and rheological properties through its oxygen-containing functional groups while also contributing to mechanical reinforcement and interlayer adhesion [52,53]. However, carbon nanotubes (CNTs), despite their exceptional tensile strength and electrical conductivity, often suffer from aggregation and poor dispersion, which can lead to nozzle clogging and inconsistent filament formation [54,55]. ZnO NPs offer a balanced approach, modestly improving viscosity, mechanical stability, and antibacterial functionality, although their effects on print fidelity are less pronounced compared to silicates [55]. Ag NPs, while highly effective for imparting antimicrobial properties, generally have limited influence on rheological behavior and structural stability unless incorporated into hybrid systems with polymers or other nanofillers [56,57]. Studies have shown that silicates and functionalized graphene are most effective in enhancing both print fidelity and structural stability in extrusion-based 3D printing systems.

### 3.2. Cell Viability, Proliferation, and Differentiation

**Figure 6:**
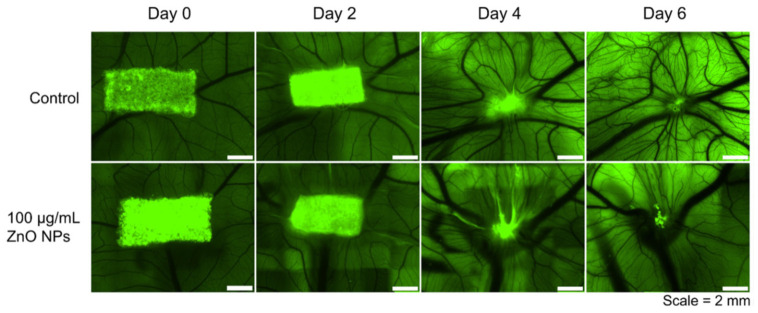
Representative Images of the Development of the Chorioallantoic Membrane treated with ZnO NPs Applied onto a Gelatin Sponge. The vasculature of the chorioallantoic membrane developed rapidly from EDD 8 (day 0) to EDD 14 (day 6) (first row). Treatment with 100 µg/mL ZnO NPs onto an auto fluorescent gelatin sponge (in green) did not influence the development (second row). The gelatin sponge is gradually degraded by the highly vascularized CAM over time. There were no significant negative reactions observed following treatment with ZnO NPs. Reproduced with permission from [58], MDPI, 2021, under CC by 4.0 license.

Cellular responses in 3D bioprinted nanocomposite systems are strongly influenced by the type of incorporated nanomaterial, particularly with respect to cell viability, proliferation, and lineage-specific differentiation, Table 5. Silver nanoparticles (AgNPs) are widely used for their strong antimicrobial activity; however, their biological effects are highly dose-dependent. While low concentrations can maintain acceptable cell viability, higher loadings often induce cytotoxicity due to Ag^+^ ion release and reactive oxygen species (ROS) generation, which can limit long-term cell proliferation in bioprinted constructs [57,58,59]. In contrast, ZnO NPs generally exhibit a more balanced biological profile, supporting enhanced cell adhesion and proliferation at optimized concentrations while promoting osteogenic differentiation through Zn^2+^-mediated signaling pathways; however, excessive ZnO content may still lead to cytotoxic effects [55,56], Figure 6.

Among the nanomaterials studied, nano silicates and bioactive glass demonstrate the most favorable overall cellular response, as their controlled ionic dissolution (e.g., Si, Mg, Li) enhances extracellular matrix (ECM)-like interactions, thereby improving cell viability, proliferation, and osteogenic or chondrogenic differentiation in 3D printed hydrogels [50,51]. Carbon-based nanomaterials, particularly graphene and graphene oxide, further contribute to improved cell proliferation and differentiation by enhancing electrical conductivity and providing favorable cell–material interactions; graphene has been shown to promote neural and osteogenic differentiation when properly dispersed within bioinks [52,53]. However, CNTs, although mechanically and electrically superior, often present dispersion-related challenges and potential cytotoxicity if not adequately functionalized, which can negatively affect cell viability [54,55].

### 3.3. Mechanical Performance of Printed Constructs

The mechanical performance of 3D bioprinted constructs is strongly governed by the type of nanofiller incorporated into the bioink, as each system contributes differently to stress transfer, network formation, and structural integrity, Table 6.

**Figure 7:**
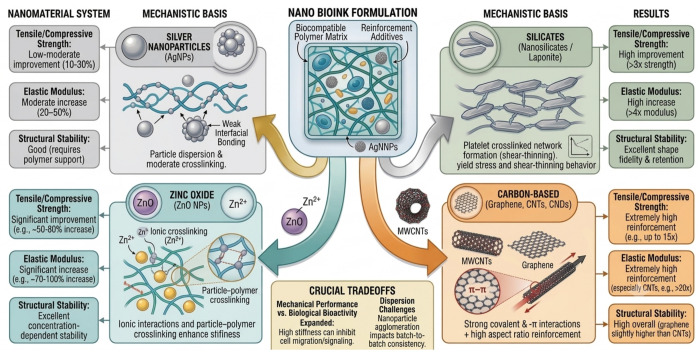
Illustrative representation of comparative properties of different nano bioinks. The figure was created with assistance from Google Gemini and subsequently reviewed, edited, and scientifically validated by the authors.

Ag NPs generally provide only limited mechanical reinforcement, as they behave primarily as inert fillers with weak interfacial bonding to polymer matrices. As a result, improvements in tensile or compressive strength are typically modest and strongly dependent on the presence of additional reinforcing polymers or crosslinkers [56,57]. In contrast, ZnO NPs offer a more pronounced strengthening effect due to ionic interactions and partial coordination with functional groups in hydrogel networks, which enhances stiffness and improves overall construct stability; however, this effect is concentration-dependent and may plateau or reverse at higher loadings due to particle aggregation [58,59]. Among the systems considered, silicate-based nanomaterials such as nano silicates and Laponite exhibit the most effective mechanical reinforcement in 3D bioprinted scaffolds. Their platelet-like morphology and charged surfaces enable the formation of physically crosslinked, percolated networks within hydrogel matrices, resulting in significantly improved yield stress, shape fidelity, and compressive modulus. These structural interactions make silicates particularly effective in maintaining printed architecture under physiological conditions [50,51]. CNTs provide the highest intrinsic mechanical reinforcement due to their exceptional stiffness and aspect ratio. Graphene enhances load transfer across the matrix through strong interfacial interactions and planar reinforcement, while CNTs contribute high tensile strength along their axial direction. However, their effectiveness is often limited by dispersion challenges, agglomeration, and inconsistent distribution within bioinks, which can reduce overall mechanical uniformity in printed constructs. Overall, silicates offer the most reliable improvement in print stability and structural fidelity, whereas carbon-based nanomaterials provide the greatest theoretical reinforcement potential, albeit with greater processing complexity. The comparative properties are illustrated in Figure 7.

### 3.4. Long-Term Biocompatibility and Integration

Long-term biocompatibility and successful integration of 3D bioprinted constructs depend on the ability of incorporated materials to maintain cellular compatibility, avoid chronic inflammatory responses, and support stable tissue remodeling over time. Ag NPs exhibit strong antimicrobial activity; however, prolonged exposure can lead to persistent ion release, oxidative stress, and potential cytotoxicity, which may compromise long-term tissue integration [56,57]. ZnO NPs generally show improved biocompatibility at optimized concentrations, as released Zn^2+^ ions can support enzymatic activity and tissue regeneration, particularly in bone-related applications; nevertheless, excessive accumulation may still induce inflammatory responses [55,58]. Silicate-based nanomaterials such as nano silicates and Laponite demonstrate superior long-term integration due to their controlled ionic dissolution (e.g., Si, Mg, and Li), which promotes extracellular matrix (ECM) deposition, vascularization, and sustained cell–material interactions, thereby enhancing tissue remodeling and scaffold integration [50,51]. Carbon-based nanomaterials, particularly graphene and graphene oxide, have also shown promising long-term biocompatibility when properly functionalized, as they support stem cell adhesion, proliferation, and lineage-specific differentiation without significant chronic toxicity at low concentrations; however, CNTs require careful surface modification to minimize aggregation and inflammatory responses that may hinder long-term integration [52,53,54].

## 4. Application-Oriented Nano Bioink Design

### 4.1. Nano Bioinks for Skin and Wound Healing Applications

Skin and wound healing represents one of the most clinically compelling application domains for nano bioink-based 3D bioprinting, as the hierarchical architecture of native skin tissue comprising stratified epidermal layers, a collagen-dense dermis, and a highly vascularized subdermal matrix demands bioink systems that simultaneously provide mechanical support, antimicrobial protection, pro-angiogenic signaling, and controlled moisture retention [37]. Among the nano bioinks, Ag NP bioinks are arguably the most directly relevant to wound healing applications, given the well-established role of silver as a broad-spectrum antimicrobial agent in clinical wound management. Ag NP-loaded alginate/nanocrystalline cellulose (ALG/CNC) scaffolds have demonstrated potent antibacterial activity against both *Staphylococcus aureus* and *Pseudomonas aeruginosa*—the two most prevalent organisms implicated in chronic wound infection—while maintaining structural integrity and acceptable cytocompatibility at controlled nanoparticle loadings [37]. Minimum inhibitory concentrations in the range of approximately 10 µg/mL have been reported for Ag NP containing composite scaffolds against both Gram-positive and Gram-negative strains, suggesting that infection-resistant wound dressings can be produced at nanoparticle concentrations that do not compromise cell viability [40]. ZnO NPs offer a complementary profile for wound healing applications, combining antibacterial activity with osteogenic potential and the capacity to support keratinocyte and fibroblast proliferation—cell types central to epidermal regeneration and dermal remodeling. ZnO-reinforced alginate hydrogels at concentrations of 0.5–1 wt.% provided significant antibacterial efficacy while maintaining cell viability above 85%, a threshold widely cited as indicative of acceptable cytocompatibility [41]. Furthermore, gelatin-ZnO hydrogel systems have shown tensile strength improvements of up to 67% over non-reinforced controls [43], which is particularly relevant to wound healing scaffolds that must resist mechanical disruption at the wound site during patient movement and dressing changes. Nanosilicate-based bioinks, particularly hydrogel formulations with Laponite, contribute to wound healing through a distinct and complementary mechanism: their controlled ionic dissolution releases biologically active Si, Mg, and Li ions that promote extracellular matrix (ECM) deposition, stimulate vascularization, and enhance fibroblast-mediated tissue remodeling—processes that are rate-limiting in chronic wounds and diabetic ulcers [50,51]. The ability of nano silicate bioinks to act as sustained-release reservoirs for pro-angiogenic growth factors further distinguishes them from conventional wound dressings, enabling spatially controlled vascularization within bioprinted skin constructs [18]. In contrast, carbon-based nanomaterials—graphene oxide, carbon nanotubes, and carbon nanodots—have a more limited direct role in skin and wound healing bioprinting, primarily because their principal functional advantages, including electrical conductivity enhancement and stem cell differentiation, are less relevant to the predominantly mechanical, antimicrobial, and angiogenic requirements of wound repair. However, reduced graphene oxide (rGO) has shown moderate antibacterial activity through membrane interaction mechanisms, and carbon nanodots have demonstrated mild antimicrobial behavior associated with reactive oxygen species (ROS) generation [49], suggesting that these materials may find utility in wound healing formulations designed for infected or oxidatively stressed wound microenvironments. More broadly, multi-component nano bioink systems that combine the antimicrobial efficacy of Ag NPs or ZnO NPs with the rheological properties of nano silicates within a hydrogel matrix, such as gelatin methacrylate (GelMA) or alginate, represent a particularly promising design strategy for bioprinted wound dressings capable of addressing the multifactorial challenges of chronic wound management.

### 4.2. Bone and Cartilage Tissue Engineering

Bone and cartilage tissue engineering represent major application areas for nano bioink development because both tissues require biomaterials that can balance printability, cytocompatibility, mechanical integrity, and lineage-specific biological signaling. Bone tissue is highly mineralized, vascularized, and mechanically rigid, whereas cartilage is avascular, hydrated, and mechanically resilient, creating distinct design requirements for bioinks intended for skeletal tissue repair. Conventional hydrogel bioinks often support cell viability but lack sufficient mechanical strength, osteoconductivity, and long-term structural stability for bone and osteochondral applications [60,61,62,63]. The incorporation of nanoscale components such as hydroxyapatite, bioactive silica nanoparticles, nano silicates, and graphene oxide has therefore emerged as a promising strategy to improve both the biological and physicochemical performance of printed bone and cartilage constructs [64,65,66,67,68].

For bone tissue engineering, nano bioinks are commonly designed to recapitulate aspects of the native mineralized extracellular matrix by incorporating calcium phosphate-based materials, hydroxyapatite, or silicate-based nanomaterials into hydrogel networks [65,66]. Hydroxyapatite-containing GelMA bioinks are particularly relevant because hydroxyapatite provides osteoconductive cues while GelMA supports cell encapsulation, photo crosslinking, and extrusion-based bioprinting. Jahangir and colleagues developed a dual-bioink system using osteoblast-laden GelMA/hydroxyapatite for the bone-like phase and chondrocyte-laden tyraminated hyaluronic acid for the cartilage-like phase, demonstrating the value of multiphasic formulations for osteochondral tissue engineering [66]. Bioactive silica nanoparticle-containing GelMA bioinks have also shown strong potential for bone regeneration by supporting extrusion printability, nanoparticle dispersion, and osteogenic differentiation of human bone marrow-derived mesenchymal stromal cells [64]. These findings support the use of bioactive ceramic and silica-based nanophases as osteoinductive components in cell-laden nano bioinks for bone repair [65,66]. Nano silicate containing bioinks are especially important for skeletal applications because they can simultaneously improve rheological behavior, structural fidelity, protein retention, and cell-instructive bioactivity [64]. Cidonio and colleagues [64] demonstrated that Laponite–GelMA nanocomposite bioinks improved shape fidelity and interconnected porosity in extrusion-bioprinted fibers while supporting osteogenic and angiogenic tissue formation. This is particularly relevant to bone tissue engineering because successful bone regeneration requires not only osteogenic differentiation but also vascularization to support nutrient transport, matrix remodeling, and tissue integration [64,69].

Graphene oxide-based nano bioinks have also shown promise for bone tissue engineering because graphene oxide can improve printability, mechanical performance, and cell-material interactions when incorporated at optimized concentrations [67]. Zhang and colleagues [67] developed a human mesenchymal stem cell-laden graphene oxide/alginate/gelatin composite bioink and reported that graphene oxide improved bioprintability, scaffold fidelity, compressive modulus, and early cell viability in bone-mimicking constructs. However, graphene oxide concentration must be carefully optimized because excessive loading may alter swelling behavior, mechanical uniformity, or long-term matrix remodeling. This concentration-dependent response is particularly relevant for nano bioink design because nanomaterials can improve scaffold function at low or moderate doses while creating aggregation, cytotoxicity, or reproducibility issues at higher concentrations [67].

For cartilage tissue engineering, nano bioinks must support chondrocyte or mesenchymal stem cell viability while promoting chondrogenic differentiation and deposition of cartilage-specific extracellular matrix components such as glycosaminoglycans and type II collagen [63,67]. Hydrogel-based bioinks are widely used for cartilage applications because their high-water content and soft matrix properties partially resemble the native cartilage microenvironment. However, many hydrogel bioinks require reinforcement to achieve the mechanical performance needed for articular cartilage repair, particularly under compressive loading [63]. Graphene oxide-doped GelMA/PEGDA bioinks have been shown to promote chondrogenic differentiation of human bone marrow mesenchymal stem cells in 3D bio printed scaffolds, supporting the role of carbon-based nanomaterials in cartilage-oriented biofabrication. The ability of graphene oxide to influence cell adhesion, matrix deposition, and lineage-specific differentiation makes it a valuable nanomaterial for cartilage and osteochondral bioink formulations [67].

Overall, bone and cartilage nano bioinks should be designed according to the distinct functional requirements of each tissue rather than treated as a single musculoskeletal application [62,63]. Bone-oriented nano bioinks benefit from osteoconductive and angiogenic components such as hydroxyapatite, bioactive silica, nano silicates, and vascular-patterned bioinks [64,65,66,67,68,69]. Cartilage-oriented nano bioinks require hydrated, cytocompatible, mechanically reinforced matrices capable of supporting chondrogenesis and cartilage-specific extracellular matrix deposition [63,67]. Osteochondral repair may require multiphasic or gradient bioinks that integrate a mineralized bone-like region with a softer cartilage-like region, allowing spatially controlled support for osteogenesis and chondrogenesis within a single engineered construct [66]. Future nano bioink development for skeletal tissue engineering should prioritize controlled nanomaterial dispersion, tissue-specific mechanical tuning, long-term matrix remodeling, and reproducible biological outcomes across laboratories [62,64,66,67].

### 4.3. Drug Delivery Applications

For drug delivery applications, nanoparticle-loaded bioinks provide sustained and spatially controlled release of therapeutic agents, while organ and tissue systems fabricated with nano bioinks offer physiologically relevant platforms for drug screening and toxicity testing, potentially reducing reliance on animal models [69,70,71,72,73,74,75,76,77,78,79,80,81,82,83,84,85,86,87,88,89]. The variability of nanoparticle composition and surface chemistry allows for control over release kinetics. This mimics the dynamic biochemical environments encountered in living beings. In addition to nano particles, bioinks laden with microspheres are also used in drug delivery applications. One study reported that microspheres containing gulsterone in bioinks can aid in the differentiation of human induced pluripotent stem cells into other cell types [88]. This creates an avenue for the creation of multicellular structures that are incredibly complex. Another approach uses nano silicate based bioinks, which are capable of separating protein therapeutics within 3D-printed structures for long periods of time. This enables the sustained release of pro-angiogenic growth factors that promote vascularization within the scaffold [88]. This ability for localized, long-term protein delivery directly within a bio printed construct represents a significant advantage over conventional drug delivery systems, which often lack the precision needed to guide complex tissue regeneration.

### 4.4. Neural and Cardiac Tissue Regeneration

In regenerative medicine, nano bioinks enable the fabrication of biomimetic scaffolds that simulate and enhance the nanoscale architecture and biochemical composition of native extracellular matrix, promoting enhanced cell adhesion, proliferation, and differentiation [79]. A common drawback of bioinks used as artificial scaffolds for tissue growth is mechanical strength. The strength and tunability of a bioink scaffold can be assisted using nano ceramics. Hydroxyapatite, specifically, can be utilized as a scaffold-bearing material. Including hydroxyapatite nanoparticles into a polyurethane-based bioink is shown to increase the modulus of compression of the resulting bioink [80,81,82]. Calcium phosphate nano ceramics can reportedly increase the differentiation of mesenchymal stem cells, aiding in the regeneration of bone tissue [82]. When these nanoceramics are integrated with a bio printed scaffold, they can enhance the human body’s already impressive regenerative capabilities. Neural and cardiac tissues present some of the most demanding engineering challenges in regenerative medicine. Both require scaffolds that simultaneously provide biomechanical support, cellular compatibility, and electrical conductivity at physiologically relevant levels. Among nano bioinks, graphene and carbon-based materials, in particular graphene oxide (GO), reduced graphene oxide (rGO), and carbon nanotubes (CNTs), are the most directly and extensively relevant to neural and cardiac tissue bioprinting owing to their exceptional electrical conductivity, mechanical reinforcement capability, and documented capacity to support the maturation and functional coupling of electroactive cell types [70]. In contrast, AgNP-based and ZnO NP bioinks have limited roles in neural and cardiac tissue engineering, as they do not address the electrical signaling requirements central to both neural and cardiac tissue function. GelMA- and alginate-based bioinks incorporating rGO produce conductive printed constructs that support cardiomyocyte synchrony, sarcomere development, and gap junction formation. These structural features are markers of electromechanical maturation and are largely absent in cells cultured on non-conductive substrates [71,72,73]. Shin and colleagues developed [74] a composite bioink combining porcine cardiac decellularized extracellular matrix (cdECM) with Laponite-XLG nanoclay and poly(ethylene glycol)-diacrylate (PEG-DA). In this system, the Laponite component enhanced viscosity and shear storage modulus to enable high-resolution extrusion-based printing, while PEG-DA provided post-print photopolymerization for structural stabilization; encapsulated human cardiac fibroblasts demonstrated greater than 97% viability after 7 days, confirming the cytocompatibility of the cdECM/nano silicate composite formulation. For neural tissue engineering, GO-incorporated GelMA hydrogels have been shown to overcome the intrinsically poor electroactive properties of conventional hydrogel bioinks by simultaneously enhancing electrical conductivity and mechanical stiffness within a single formulation suitable for extrusion-based bioprinting [70,71,72,73]. GO’s oxygen-containing functional groups promote uniform dispersion within aqueous hydrogel systems, improving shear-thinning behavior and print resolution while also providing surface chemistry that supports neural stem cell adhesion and differentiation toward neuronal lineages [72,73,74]. CNT-based bioinks and scaffolds have similarly demonstrated the capacity to promote neuronal growth and electrophysiological development through their large negatively charged surface area, which facilitates interactions with ionic species and adsorption of bioactive molecules critical to neurotrophic signaling [75]. CNTs have been incorporated into silk fibroin/GelMA/PEG composite bioinks to produce conductive constructs that support both neural and cardiomyocyte differentiation, though CNT purity and concentration remain significant challenges, limiting reproducibility across studies [75]. The most promising design direction for neural and cardiac nano bioinks involves an integrated approach that combines the printability advantages of nano silicates, the electrical performance of rGO or functionalized CNTs, and the biological signaling of dECM-derived or growth factor-loaded matrices.

### 4.5. Tumor Models and Disease-on-a-Chip Systems

The applications of nano bioinks span regenerative medicine, drug delivery systems, disease modeling, and biosensing platforms. Three-dimensional tumor models in vitro (e.g., tumor spheroids, tissue-derived tumor spheres, organotypic multicellular spheroids, and 3D scaffolds) mimic the in vivo micro-environment better than 2D cultures because they enable understanding of cancer cell interactions, biological processes, architecture, and drug-screening effects, despite their significant limitations, and are well detailed in the literature [76]. Gelatin, collagen, cellulose, alginate, chitosan, hyaluronic acid, decellularized extracellular matrix (dECM), agarose, fibrinogen, poly(ethylene Glycol) PEG, and Pluronic are the most common hydrogels used for 3D bioprinting in cancer research [76,78]. Addition of nanomaterials such as nano clays or nanocrystals to any of these hydrogels makes them nano bioinks with enhanced physical properties, for example, improved rheology, porosity, and printability. The gold standard extracellular matrix (ECM) for 3D tumor models has been Matrigel, a basement membrane made of a gelatinous thermo-sensitive protein mixture derived from Engelbreth-Holm-Swarm mouse sarcoma tumors that contains mainly proteins (laminin, type IV collagen, and entactin) and a small portion of proteoglycans and growth factors [77]. Matrigel offers powerful advantages over other materials, including key features of a tumor microenvironment: recapitulation of metabolism by providing and permitting near-ideal diffusion of nutrients and growth factors and cellular layer structural integrity (outer core proliferating, middle region quiescent, and inner necrotic core) [78]. However, major disadvantages include its high price, its mouse origin, and an undefined chemical composition that varies by batch. Due to this, there is a need for improved hydrogels that can serve as a base material for bioinks, which are the complete formulation that goes into the 3D bioprinter nozzle, consisting of living cells and, for example, a hydrogel. Gelatin methacryloyl, or methacrylate (GelMA), a thermosensitive compound, is one of the best-studied potential alternatives to Matrigel for 3D culturing because of its controllability in terms of methacryloyl functionalization and physicochemical properties that enable improved batch-to-batch consistency compared to Matrigel [77]. Cheng et al. demonstrated [79] the superiority of 3D models constructed by bioprinting a tumor-vessel-bone metastasis system using GelMa-based photo-cross-linkable bioinks containing breast cancer cells (MDA-MB-231), human umbilical vein endothelial cells (HUVECs), and osteoblasts (h-OB). They were able to imitate native metastatic niches with vascularized tumors and bone tissue. Their study established a powerful platform for testing drugs that inhibit cancer proliferation and metastasis and can provide insight into the drug mechanism of action because their co-culture models closely recapitulate in vivo tumor metastasis microenvironment cues. GelMa combined with photoinitiator LAP (lithium phenyl-2,4,6-trimethylbenzoylphosphinate) was used with dielectrophoretic droplet manipulation into a 3D bioprinting system to induce the in-situ formation of compact, viable, and uniform tumor spheroids to build customizable, biomimetic tumor microenvironments [80,81,82,83].

Organ-on-a-chip systems converge 3D bioprinting with microfluidics to replicate the structure and function of human organs in vitro in miniature scale for advancing disease modeling [83,84]. The microfluidics recreate the dynamic function of media flow, shear stress, and pressure, which are key physiological characteristics of a tissue microenvironment, for example, enabling perfusion within a tumor microenvironment model. Tumor-on-a-chip systems can be used for screening multiple cancer patient tissue samples, drug screening, tumor precision diagnosis, and studying cancer cell migration and invasion properties [84]. The on-chip application allows for versatile real-time monitoring of physicochemical phenomena. James et al. demonstrated how electrophysiological measurements and microelectrode architecture can be applied to acquire metabolic information for 3D mitochondrial biosensing [85].

## 5. Challenges in Translation and Clinical Implementation

Biocompatibility, non-immunogenicity, and biodegradability are some of the limitations of bioinks. A solution proposed by Wang et al. [86] was to use covalently bonded hyaluronic acid-gelatin and reversible-crosslinked gelatin-dextran with tunable viscoelasticity to mimic natural extracellular matrix microenvironments, strengthening cell-matrix interactions for organoid culture and modeling tumor metastasis [86]. Lack of oxygenation, leading to necrosis, within 3D tissue spheroid models is another significant limitation, making size consistency a critical parameter for evaluating drug penetration and transport in any 3D tumor model [76]. The ideal size to prevent hypoxia due to lack of vascularization is a diameter smaller than 300 μm [77]. A large diameter or surface area can complicate drug penetration and delivery, limiting interaction between drugs and cells within the 3D environment [76]. Beyond size, Badea et al. [78] describe several additional critical limitations, including limited standardization and analysis of optimized protocols for 3D cultures, and note the difficulty of analyzing gel-laden 3D constructs when cells must be extracted from the spheroids [77].

### 5.1. Nanotoxicity and Biosafety Concerns

Nanotoxicology (nano safety) research has been described at length by others who demonstrated the necessity of combining advanced strategies such as predictive toxicology, high-throughput screening (HTS), nanomaterial library, multi-omics approach, adverse outcome pathways, in silico analysis and data mining, and “safe-by-design” for safe translational use [87,88]. Engineered nanomaterials (ENMs) that induce nanotoxicity create significant clinical translational barriers in the development of nano bioink applications [88]. Assessment methods that combine biological functions, including metabolomics, proteomics, and physicochemical assessments, are necessary. A powerful example was shown by Cai et al. [88], developing a multi-hierarchical nano-SAR profile that also provided mechanistic insights from the relationships between seven physicochemical properties of their nanoparticles (diameter/length, thickness, aspect ratio, surface area, Zeta potential, hydrodynamic size, and surface reactivity) and their bio-effects (inflammation, and energy metabolism, cell proliferation, protein biosynthesis, lipid metabolism, cell migration, global change) [88]. Nano bioinks possess physicochemical properties—including extreme surface area-to-volume ratios, surface charge, redox activity, and ionic dissolution behavior that are not predictable and that can produce biological effects disproportionate to their mass fraction within a formulation [89]. Critically, the toxicological profile of nano bioinks differs substantially in mechanisms, target-organ specificity, and concentration dependence. Ag NPs present the most extensively characterized and clinically consequential nanotoxicity profile. The primary mechanism of Ag NPs cytotoxicity is dual: ionic, through sustained release of Ag^+^ ions that disrupt mitochondrial membrane potential, activate caspase-mediated apoptotic cascades, and generate reactive oxygen species (ROS); and particulate, through direct physical perturbation of membranes and intracellular oxidative damage [90]. However, the long-term cumulative effects of sustained Ag^+^ release from implanted bioprinted scaffolds have not been prospectively characterized in large-animal or human studies, representing a critical data gap for clinical translation. ZnO NPs exhibit a nanotoxicity profile that is mechanistically similar to Ag NPs but operates through Zn^2+^ ion dissolution rather than Ag^+^ release. ZnO NPs induce hepatotoxicity, nephrotoxicity, and pulmonary injury through a convergence of ROS-mediated oxidative stress, mitochondrial dysfunction, lipid peroxidation, and depletion of antioxidant enzymes, including superoxide dismutase (SOD) and glutathione (GSH). For bioink applications, these findings collectively indicate that ZnO NP concentration must be rigorously optimized within a narrow therapeutic window, which is typically reported as 0.5–1 wt.% in alginate hydrogel systems, to preserve cell viability above 85% while maintaining antimicrobial efficacy [41].

Nano silicates, particularly synthetic Laponite, represent the nanomaterial class with the most favorable overall biosafety profile, though important concentration-dependent concerns remain. Laponite degrades through hydrolysis of its silicate layers in aqueous physiological environments, releasing biologically tolerated ions—primarily Si(OH)_4_, Mg^2+^, and Li^+^—that at low concentrations are cytocompatible and, in the case of silicic acid, have been shown to stimulate osteogenic gene expression and ECM deposition [91]. In vivo studies using Laponite-composite bone graft systems and gelatin/Laponite embolic agents have reported no foreign body response, no significant inflammation, and hemocompatibility consistent with acceptable clinical safety profiles [92]. However, cytotoxic and genotoxic effects of Laponite on murine adipose-derived stem cells have been documented at elevated concentrations, with the extent of DNA damage correlating with Laponite dose and exposure duration [91]. The high ionic surface charge of Laponite, which is central to its rheological and biological factor-retention functionality, can also promote non-specific protein adsorption and complement activation at high concentrations—potential immunological concerns that are incompletely addressed by current preclinical testing protocols [92]. Carbon-based nanomaterials—graphene oxide (GO), reduced graphene oxide (rGO), carbon nanotubes (CNTs), and carbon nanodots (CNDs) present the most heterogeneous and context-dependent nanotoxicity profile. Carbon nanodots occupy the most favorable end of the carbon nanomaterial biosafety spectrum, exhibiting low cytotoxicity, efficient cellular uptake, and negligible acute inflammatory response at biomedically relevant concentrations, though batch-to-batch quantum yield variability and inconsistent characterization protocols limit the reproducibility of safety assessments across studies [90].

### 5.2. Scalability and Manufacturing Challenges

Scale-up production methods are costly due to the high cost of materials for maintaining cell viability, aseptic manufacturing, current Good Manufacturing Practices (cGMP) compliance, lack of high-throughput scaffold fabrication processes amenable to scale-up, and the complexity of bioreactor systems [93]. The complexity of combinations of variables, including but not limited to biomaterial selection, printing resolution, and bioprinting modality, along with cell viability, is a significant challenge, but these will be overcome with increasingly accurate models based on artificial intelligence learnings. Another significant manufacturing challenge is the destructive nature of the analyses required for quality assessment, manufacturing scale-up, and the translation of 3D construct technologies. Though significant efforts, including dielectric impedance spectroscopy (DIS) and application of supervised machine learning, are being developed to overcome these challenges [94,95].

### 5.3. Regulatory Considerations for Nano Bioinks

Ethical considerations surrounding the use of nano bioinks, including patient consent for bio printed implants containing nanomaterials, equitable access to advanced regenerative therapies, and environmental implications of nanomaterial production and disposal, have received limited attention in the literature to date. This review aims to provide a comprehensive analysis of current nano bioink fabrication strategies, survey the diverse applications across regenerative medicine, drug delivery, and biosensing, and critically examine the key technical, regulatory, and ethical challenges that must be addressed to realize the full translational potential of these advanced materials. Regulatory agencies like the Food and Drug Administration (FDA) and the European Medicines Agency (EMA) promote safety and efficacy in clinical applications backed by rigorous scientific data. Supporting data generation with regulatory oversight requires integrating advanced expert insight into clinical frameworks. This integration must be grounded in the quantitative measurement of nanotoxicological mechanisms responsible for short- and long-term adverse patient outcomes [93,94,95]. Although nano-bioinks have demonstrated tremendous potential in tissue engineering and regenerative medicine, their clinical translation remains constrained by regulatory uncertainty, safety concerns, and manufacturing challenges. Because nano-bioinks integrate living cells, biomaterials, bioactive molecules, and nanomaterials within a single construct, they are generally regarded as combination products, requiring evaluation of both biological and device components. This substantially complicates regulatory approval compared with conventional biomaterials [96]. The regulatory pathway depends on the composition and intended clinical application of the nano-bioink. In the United States, products containing cells and nanomaterials may require coordinated evaluation by the Center for Biologics Evaluation and Research (CBER), the Center for Devices and Radiological Health (CDRH), and, in some cases, the Center for Drug Evaluation and Research (CDER). Depending on product classification, developers may need to submit an Investigational New Drug (IND) application or an Investigational Device Exemption (IDE) before initiating clinical studies. Similarly, within the European Union, cell-laden bioprinted constructs are commonly regulated under the Advanced Therapy Medicinal Products (ATMP) framework or the Medical Device Regulation (MDR), requiring comprehensive preclinical characterization, Good Manufacturing Practice (GMP) compliance, and long-term safety evaluation before commercialization [97].

Safety evaluation of nano-bioinks extends beyond conventional biocompatibility testing because nanomaterials may exhibit size-dependent physicochemical and biological properties. Regulatory agencies therefore require detailed characterization of nanoparticle size distribution, morphology, surface chemistry, zeta potential, degradation kinetics, aggregation behavior, and batch-to-batch reproducibility. Furthermore, cytotoxicity, immunogenicity, hemocompatibility, genotoxicity, biodistribution, degradation products, and long-term in vivo responses must be thoroughly assessed to ensure clinical safety [98].

Manufacturing remains one of the most significant barriers to clinical translation. Most nano-bioinks are synthesized at laboratory scale, where maintaining consistent nanoparticle characteristics, rheological properties, cell viability, and printability is relatively straightforward. However, scaling production while preserving uniform particle size, dispersion stability, surface functionalization, and biological performance remains challenging. Minor manufacturing variations can substantially alter print fidelity, degradation behavior, mechanical properties, and cellular responses, making reproducibility a major regulatory concern. Another major challenge involves compliance with Good Manufacturing Practice (GMP) requirements. Industrial production demands validated manufacturing protocols, sterile processing, contamination control, traceability, in-process quality monitoring, and rigorous quality assurance systems. Sterilization methods such as gamma irradiation, ethylene oxide treatment, or steam sterilization may adversely affect nanoparticle integrity, hydrogel crosslinking, and cell viability, further complicating manufacturing. Additionally, patient-specific bioinks and point-of-care bioprinting introduce new regulatory questions regarding decentralized manufacturing, quality control, and product consistency [99]. Future progress toward clinical translation will require internationally harmonized standards for nano-bioink characterization, standardized quality-control protocols, scalable GMP-compliant manufacturing, and regulatory frameworks specifically addressing multifunctional bioprinted products. Recent initiatives such as ASTM F3659 (Standard Guide for Bioinks Used in Bioprinting) represent important steps toward establishing standardized testing methods for bioinks and facilitating regulatory approval [100].

### 5.4. Standardization and Reproducibility Issues

Standardization and reproducibility remain major challenges in nano bioink research because the final biological and structural performance of a printed construct depends on multiple interconnected variables, including polymer composition, nanomaterial concentration, cell density, rheological behavior, printing parameters, crosslinking method, and post-print culture conditions [95,96]. Even when the same bioink formulation is used, differences in printer hardware, nozzle geometry, extrusion pressure, printing speed, temperature control, and operator handling can generate substantial variability in filament formation, shape fidelity, cell viability, and construct architecture [101,102,103]. A recent round-robin study involving multiple academic laboratories demonstrated that extrusion-based printing outcomes can vary across sites despite efforts to standardize the printing process, emphasizing the need for harmonized protocols and quantitative performance metrics [101]. These findings are particularly relevant for nano bioinks because nanoscale additives can improve bioink functionality while also introducing additional sources of variability related to particle dispersion, aggregation, surface chemistry, and batch-to-batch material differences [103,104,105,106].

Rheological characterization is one of the most important requirements for improving reproducibility in extrusion-based bioprinting because bioinks must flow through the nozzle under applied shear while recovering rapidly after deposition to maintain the printed geometry [103,104,105,106]. Key rheological parameters such as viscosity, shear-thinning behavior, yield stress, storage modulus, loss modulus, and recovery behavior directly influence printability, filament continuity, construct stability, and cellular stress during extrusion [101,102,103,104,105]. Increasing viscosity or nanomaterial content may improve shape retention, but excessive viscosity can require higher extrusion pressures and increase shear-induced damage to encapsulated cells [103,104,105]. Therefore, nano bioink studies should report both material properties and printing conditions, including temperature, nozzle diameter, extrusion pressure or flow rate, printing speed, crosslinking conditions, and post-print stabilization methods [101,102,103,104,105]. Without these details, it becomes difficult to compare results across studies or reproduce reported improvements in print fidelity, mechanical strength, and cellular behavior [105].

Nanomaterial characterization is also essential for reproducibility because nanoparticle size, morphology, charge, concentration, purity, functionalization, and dispersion state can significantly affect both bioink printability and cellular responses [101,102,103,104,105]. For example, nanomaterials such as nano silicates, graphene oxide, carbon nanotubes, hydroxyapatite, and bioactive silica can alter viscoelastic properties, mechanical reinforcement, protein adsorption, growth factor retention, and lineage-specific differentiation depending on their concentration and distribution within the hydrogel matrix. However, poorly dispersed or overloaded nanoparticles may cause nozzle clogging, heterogeneous mechanical properties, reduced cell viability, inflammatory responses, or inconsistent biological outcomes. To improve reproducibility, authors should report the nanomaterial supplier, lot number when available, particle size distribution, sterilization method, dispersion protocol, final concentration, and evidence of homogeneous incorporation into the bioink. These details are especially important for nano bioinks because small changes in nanoparticle preparation can produce disproportionate effects on rheology, printability, cytocompatibility, and long-term construct performance [101,102,103,104].

Another concern of reproducibility is the lack of consistent quantitative criteria for defining printability and shape fidelity in bioprinting studies [105]. Printability is often described qualitatively, but reproducible comparison requires measurable outcomes such as filament diameter, pore size accuracy, strand spreading, layer collapse, angular accuracy, construct height retention, and deviation from the intended computer-aided design model [103,104,105]. Schwab and colleagues emphasized that printability should be assessed using both qualitative and quantitative methods because visual inspection alone is insufficient to determine whether a bioink can reliably generate complex tissue-like architectures [105]. This is particularly important for nano bioinks because nanomaterials may improve visual shape retention while simultaneously affecting cell viability, degradation, swelling, or matrix remodeling over time [101,102,103,104,105]. Therefore, reproducibility should be evaluated not only immediately after printing but also after crosslinking, culture, degradation, and tissue maturation phases [101,102,103,104,105].

Biological reproducibility is equally important because nano bioinks are intended to support living cells rather than function only as printable materials. Therefore, improving standardization in nano bioink research requires integrated reporting of material formulation, nanomaterial characterization, rheological properties, printing parameters, crosslinking conditions, biological endpoints, and long-term construct performance [95,96,97,98,99,100,101,102].

## 6. Future Perspectives in Rational Nano Bioink Engineering

Despite rapid progress in nanomaterial-based bioinks and hybrid biomaterial systems, several critical gaps continue to limit their translational potential. There is a lack of standardized protocols for evaluating nanomaterial dispersion, stability, and long-term bioactivity within complex polymeric matrices, leading to poor reproducibility across studies. Most reported systems rely on single-function optimization (e.g., mechanical strength or conductivity) while neglecting the integrated balance between printability, cytocompatibility, and degradation kinetics required for in vivo applications. Mechanistic understanding of nano–biointerface interactions remains incomplete, particularly regarding how surface chemistry and particle morphology influence immune response and cellular remodeling over time. Also, the scalability remains a major bottleneck, as many fabrication strategies are confined to laboratory-scale synthesis without clear pathways for industrial-scale manufacturing under Good Manufacturing Practice (GMP) conditions.

### 6.1. AI-Assisted Nanobioink Design

Traditional bioink development often relies on time-intensive trial-and-error experimentation due to the complex interactions among rheological behavior, crosslinking kinetics, cell viability, and scaffold fidelity. AI-assisted nano bioink design has emerged as a transformative strategy in advanced bioprinting and regenerative medicine by integrating artificial intelligence (AI), machine learning (ML), and biomaterials engineering to optimize bioink composition, printability, and biological performance [106,107,108,109]. Recent studies demonstrate that AI-driven predictive models can accelerate the optimization of nano bioinks by analyzing large datasets involving viscosity, shear-thinning behavior, nanoparticle concentration, and cellular responses to identify formulations with enhanced mechanical stability and tissue compatibility [108]. Machine learning algorithms have also been used to improve extrusion precision, printing resolution, structural integrity, and post-printing maturation in 3D bioprinting systems. Furthermore, nanomaterial incorporation, including graphene oxide, nanocellulose, hydroxyapatite nanoparticles, and carbon-based nanostructures, has significantly improved conductivity, bioactivity, and cellular differentiation within bioinks. AI-guided nano bioink engineering, therefore, offers a powerful platform for designing patient-specific scaffolds, functional tissue constructs, and intelligent biomaterials with applications in wound healing, organ regeneration, and personalized medicine [108].

### 6.2. Personalized and Patient-Specific Bioinks

Personalized and patient-specific bioinks represent a major advancement in regenerative medicine and precision healthcare by enabling the fabrication of biologically tailored tissue constructs that closely mimic the patient’s native cellular and extracellular microenvironment [109]. Unlike conventional biomaterials, patient-specific bioinks are designed using autologous cells, tissue-derived extracellular matrix components, growth factors, and customized biomaterial formulations to enhance biocompatibility, minimize immune rejection, and improve tissue regeneration outcomes. Artificial intelligence and computational modeling are increasingly being integrated into bioink design to optimize rheological behavior, printability, scaffold architecture, and cellular differentiation based on patient-derived biological datasets [110]. Additionally, nanomaterial incorporation, including nanocellulose, graphene derivatives, hydroxyapatite nanoparticles, and bioactive nanocomposites, has improved mechanical strength, conductivity, and bioactivity of customized bioinks. Personalized bioink technologies, therefore, offer significant potential for creating functional tissue substitutes, accelerating wound healing, and advancing precision medicine through highly adaptive and biologically responsive engineered tissues [110,111].

### 6.3. Multi-Responsive Hybrid Nano Bioinks

Multi-responsive hybrid nano bioinks have emerged as a next-generation platform in tissue engineering, regenerative medicine, and biofabrication due to their ability to dynamically respond to multiple physiological and external stimuli while supporting high-resolution bioprinting and cellular functionality [112,113,114,115,116]. Unlike conventional bioinks, hybrid nano bioinks integrate biomacromolecules, nanomaterials, living cells, and bioactive molecules into multifunctional systems capable of adapting to environmental changes such as pH, temperature, light, magnetic fields, electrical stimulation, enzymatic activity, and mechanical stress [117,118]. These intelligent bioinks offer enhanced control over scaffold architecture, cell behavior, drug release, and tissue maturation, making them highly attractive for advanced biomedical applications, including neural engineering, wound healing, cartilage regeneration, biosensing, and personalized medicine.

The incorporation of nanomaterials into bioink matrices significantly improves the physico-chemical and biological performance of printed constructs. Nanoparticles such as graphene oxide, carbon nanotubes, hydroxyapatite, silica nanoparticles, gold nanoparticles, nanocellulose, and magnetic nanoparticles have been extensively investigated due to their ability to enhance conductivity, mechanical strength, rheological behavior, biocompatibility, and cellular interactions [118,119]. Conductive nanomaterials improve electrical signaling in engineered cardiac and neural tissues, while ceramic nanoparticles such as hydroxyapatite promote osteogenic differentiation and bone tissue regeneration. Nanocellulose and silica-based nanomaterials enhance print fidelity, shear-thinning behavior, and structural stability, which are critical for maintaining scaffold integrity during extrusion-based bioprinting processes.

Hybrid nano bioinks are designed to exhibit multi-responsive behavior through the synergistic interaction between smart polymers and functional nanomaterials. Thermo-responsive polymers such as poly(N-isopropylacrylamide) (PNIPAM) and gelatin methacrylate (GelMA)-based systems can undergo reversible sol-gel transitions in response to temperature fluctuations, enabling minimally invasive injectable biofabrication [112,113,114,115]. Similarly, pH-responsive hydrogels containing chitosan, alginate, or polyacrylic acid can regulate drug release and scaffold swelling behavior under acidic or alkaline conditions commonly associated with inflammation, tumor microenvironments, or infected tissues. Photo-responsive bioinks incorporating photo-cross-linkable moieties allow precise spatial and temporal control over scaffold polymerization using ultraviolet or visible light exposure. Electrically responsive nano bioinks containing conducting polymers such as polyaniline, polypyrrole, and PEDOT facilitate electrical stimulation of encapsulated cells, promoting enhanced cellular communication and tissue functionality [116,117,118,119,120].

One of the most significant advantages of multi-responsive hybrid nano bioinks is their ability to mimic the dynamic extracellular matrix (ECM) environment found in native tissues. The natural ECM continuously responds to biochemical and mechanical signals that regulate cell adhesion, proliferation, migration, and differentiation. By integrating responsive biomaterials and nanostructures, hybrid nano bioinks can provide biomimetic microenvironments that actively guide tissue development and regeneration. These systems can also support controlled and stimulus-triggered delivery of growth factors, cytokines, antimicrobial agents, and therapeutic drugs, thereby improving tissue healing efficiency and reducing systemic side effects [111,112].

Despite significant progress, several challenges remain in the development of multi-responsive hybrid nano bioinks. Achieving simultaneous optimization of biocompatibility, printability, mechanical strength, biodegradability, and responsiveness remains difficult due to the complex interplay between material components. Nanoparticle aggregation, long-term cytotoxicity, inconsistent degradation behavior, and limited scalability also hinder clinical translation. Furthermore, regulatory approval pathways for multifunctional nano bioinks remain underdeveloped because of concerns regarding nanoparticle safety, manufacturing reproducibility, and quality control. Addressing these limitations will require interdisciplinary collaboration among materials scientists, chemists, biomedical engineers, clinicians, and computational researchers.

Future developments in hybrid nano bioinks are expected to focus on self-healing biomaterials, 4D bioprinting systems, bioelectronic interfaces, and personalized regenerative therapies. Advances in stem cell engineering, nanotechnology, synthetic biology, and AI-guided biomaterials discovery will likely enable the creation of highly adaptive bioinks capable of autonomously responding to changing physiological environments. Such intelligent biomaterials may ultimately facilitate the fabrication of functional tissues and organs with unprecedented precision and biological complexity, transforming the future of regenerative medicine and personalized healthcare.

### 6.4. Translational Pathways Toward Clinical Use

The successful clinical translation of advanced biomaterials and bio fabricated tissue constructs requires a multidisciplinary framework that integrates materials science, biomedical engineering, regulatory science, manufacturing scalability, and clinical validation [117]. In the field of tissue engineering and regenerative medicine, translational pathways toward clinical use involve the systematic progression of laboratory-developed technologies from proof-of-concept studies to preclinical evaluation, regulatory approval, and eventual commercialization for patient care. Although significant progress has been made in developing smart biomaterials, nano bioinks, and bioprinted tissues, the transition from experimental research to clinical implementation remains challenging due to issues associated with reproducibility, safety, scalability, cost-effectiveness, and regulatory compliance [117].

One of the most critical steps in translational development is the optimization of biomaterial biocompatibility and biosafety. Materials intended for clinical applications must demonstrate minimal cytotoxicity, immunogenicity, and inflammatory response while supporting tissue integration and cellular functionality [118,119,120]. Hybrid nano bioinks containing nanoparticles, conductive polymers, or bioactive agents require particularly rigorous evaluation because nanomaterial accumulation and degradation byproducts may produce long-term biological effects. Therefore, comprehensive in vitro and in vivo studies are essential to assess cellular responses, biodegradation kinetics, biodistribution, and systemic toxicity prior to human application. Standardized characterization protocols and Good Laboratory Practice (GLP)-compliant testing procedures are increasingly necessary to improve reproducibility and facilitate regulatory acceptance [117].

Scalable manufacturing and process standardization also represent major translational barriers. Many biofabrication technologies currently rely on highly customized laboratory procedures that are difficult to reproduce consistently on an industrial scale. Clinical translation requires robust manufacturing platforms capable of producing biomaterials and tissue constructs with reproducible quality, structural fidelity, sterility, and functional performance. Good Manufacturing Practice (GMP)-compliant fabrication systems are therefore essential for ensuring batch-to-batch consistency and maintaining regulatory standards. Advances in automation, artificial intelligence-assisted process optimization, and closed-loop bioprinting systems are expected to improve scalability while reducing human error and manufacturing variability [117,118,119,120].

Preclinical animal studies serve as a critical bridge between laboratory research and human clinical trials. Appropriate animal models are necessary to evaluate tissue integration, vascularization, immune response, mechanical stability, and long-term therapeutic outcomes under physiologically relevant conditions. Large-animal models are particularly important for assessing scalability and surgical feasibility prior to clinical use. However, differences between animal physiology and human biology may limit predictive accuracy, highlighting the growing importance of organ-on-chip systems, computational modeling, and patient-specific in vitro platforms for translational assessment [115,116,117,118,119,120].

Translational success requires strong collaboration among academic institutions, clinicians, biotechnology companies, regulatory agencies, and healthcare systems. Intellectual property protection, technology transfer mechanisms, startup development, and industry partnerships play major roles in advancing laboratory discoveries into clinically accessible products. Economic considerations, including manufacturing cost, reimbursement policies, and healthcare accessibility, must also be addressed to ensure broad patient adoption. Continued progress in stem cell biology, nanotechnology, synthetic biology, and advanced manufacturing will likely accelerate the development of clinically viable tissue substitutes and functional artificial organs. Interdisciplinary collaboration and harmonized regulatory frameworks will remain essential for transforming experimental biomaterials research into safe, effective, and widely accessible clinical therapies.

## 7. Conclusions

In addition to the great benefits of nano bioinks, there are significant challenges that disturb the clinical translation of nano bioinks. Batch-to-batch variability in nanomaterial synthesis and bioink formulation remains a critical concern, as inconsistencies in particle size distribution, surface chemistry, and dispersion quality can negatively impact printability, mechanical properties, and biological performance. The regulatory landscape for nano bioinks is particularly complex, as these materials represent combination products that incorporate living cells, nanoscale materials with potential toxicity concerns, and biomaterials that may be subject to varying approval pathways across jurisdictions. Current regulatory frameworks, including the FDA’s guidance on combination products and the EU’s Advanced Therapy Medicinal Products (ATMP) designation, are not specifically designed to address the unique characteristics of nano bioinks, creating uncertainty regarding safety testing requirements, quality control standards, and approval pathways. Furthermore, long-term biocompatibility and potential cytotoxicity of certain nanomaterials, particularly carbon nanotubes and metal oxide nanoparticles, remain areas of active investigation and debate within the scientific community.

## Figures and Tables

**Table 1:** Comparative performance of clay-based nano bioinks.

Nano Clay Type	Structure & Composition	Key Physicochemical Properties	Role in Bioinks	Advantages	Limitations
Laponite (Synthetic Nanosilicate) [31]	Disc-shaped layered silicate (Na^+^, Mg^2+^, Li^+^)	High surface charge, shear-thinning, thixotropic behavior	Induces physical crosslinking; improves rheology	Excellent dispersion, strong rheological control, bioactive ion release	Potential cytotoxicity at high concentrations, rapid degradation in aqueous systems
Montmorillonite (MMT) [32]	Layered aluminosilicate (smectite group)	High cation exchange capacity, swelling behavior	Improves mechanical strength; acts as nanofiller; enhances polymer interaction	Abundant, cost-effective, good reinforcement capability	Poor dispersion without modification, potential aggregation
Sepiolite [33]	Fibrous magnesium silicate	High surface area, porous structure	Reinforces polymer matrix; enhances adsorption properties	High porosity, good adsorption capacity	Less explored in bioinks; dispersion challenges
Halloysite Nanotubes (HNTs) [34]	Hollow tubular aluminosilicate	High aspect ratio, lumen for loading molecules	Drug carrier; mechanical reinforcement; enhances matrix interaction	Natural nanotube structure, good loading capacity	Limited surface reactivity without modification, weaker rheological control
Bentonite [35]	Mixture rich in montmorillonite	High swelling capacity, high viscosity	Enhances viscosity and gel strength; improves print fidelity	Strong thickening agent, widely available	Can cause excessive viscosity, affecting printability
Saponite [36]	Trioctahedral smectite clay	High surface charge, layered structure	Improves rheology and crosslinking density	Similar benefits to laponite with tunable composition	Limited studies in bioprinting applications

**Table 2:** Comparative literature on Ag NPs-based bioink formulation.

Ag Nanoparticle	3D Bioprinting System	Polymer/Bioink System	Main Outcome
Ag NP hydrogel scaffold [37]	Extrusion-based 3D-printed scaffold	Alginate nanocrystalline cellulose (ALG/CNC)	Strong antibacterial activity vs. *S. aureus* and *P. aeruginosa*; improved mechanical stability
Ag/ECM hydrogels [38]	Hydrogel-based bioinks for bioprinting	ECM/synthetic hydrogel systems	Ag NPs enhance antimicrobial performance, but dispersion and cytotoxicity must be controlled
Ag composite hydrogels [39]	Bioink additives for regenerative 3D printing	Hydrogel bioinks	Ag NPs improve infection resistance in printed tissues
ECM-based hydrogels [40]	Nanomaterial-reinforced bioprinting inks	ECM-based hydrogels	Ag NPs improve functionality but require uniform dispersion

**Table 3:** Comparative literature on ZnO-based bioink formulation.

ZnO-Based Bioink	Printing System	ZnO NP Function	Key Findings
Alginate hydrogel [41]	Extrusion-based 3D printing	Antibacterial, mechanical reinforcement	ZnO (0.5–1%) improved stiffness, antibacterial activity, and maintained cell viability
PLA/ZnO nanocomposite [42]	FDM 3D printing	Mechanical, thermal enhancement	ZnO improved crystallinity, thermal stability, and enabled functional 3D printed biosensor materials
Gelatin–ZnO hydrogel [43]	Extrusion bioprinting	Antibacterial, mechanical strengthening	Tensile strength increased up to 67%, strong antibacterial activity, >85% cell viability
BT/HA + ZnO [44]	3D printed ceramic scaffold	Antibacterial, osteogenic	ZnO imparted antibacterial properties and supported bone regeneration under stimulation
ZnO biosynthesized [45]	photosynthesized	Biocompatibility, significant anticancer activity against MCF-7 breast cancer cells	ZnO NPs widely used due to low toxicity and strong biomedical compatibility

**Table 4:** Graphene-based bioink formulation.

Parameter	Graphene-Based Systems	Carbon Nanotubes (CNTs)	Carbon Nanodots (CNDs/CDs)
Typical Bioink Systems [46]	GelMA, PEGDA, polyurethane, alginate-based hydrogels	Polymer composites (PLA, PCL), hydrogel blends	Hydrogel bioinks (gelatin, chitosan, alginate)
Printability/Rheology [46]	Excellent dispersion due to oxygen functional groups; enhances shear-thinning behavior	Poor dispersion; requires surface functionalization or surfactants	Good dispersion due to small size; minimal impact on viscosity
Mechanical Reinforcement [47]	Improves stiffness, Young’s modulus, and scaffold integrity	Strong reinforcement due to high aspect ratio and tensile strength	Limited mechanical reinforcement compared to graphene/CNTs
Electrical Conductivity [48]	High conductivity (especially rGO); useful for neural/cardiac tissues	Excellent conductivity; superior for conductive scaffolds and sensors	Moderate conductivity; mainly used for sensing/imaging rather than conduction
Biological Performance [49]	Promotes stem cell differentiation (e.g., chondrogenesis, neural differentiation)	Variable biocompatibility; cytotoxicity depends on dose and purity	High biocompatibility; low toxicity and good cellular uptake
Functional Role [49]	Bioactivity, conductivity, mechanical support	Electrical conductivity, reinforcement	Bioimaging, drug delivery, fluorescence tracking
Antibacterial Properties [49]	Moderate antibacterial activity via membrane interaction	Strong antibacterial effect (mechanical disruption of membranes)	Mild antibacterial effect; mainly ROS-related
Key Applications [49]	Neural tissue engineering, cartilage regeneration, conductive scaffolds	Biosensors, conductive composites, reinforced scaffolds	Bioimaging scaffolds, smart biosensors, drug delivery
Major Limitation [47]	Potential cytotoxicity and aggregation at high loading	Agglomeration and cytotoxicity issues	Lower mechanical and electrical performance

**Table 5:** Comparison of Cell Viability, Proliferation, and mechanistic insights.

Nanomaterial System	Cell Viability	Cell Proliferation	Cell Differentiation	Key Mechanistic Insight
Silver nanoparticles (AgNPs) [58]	Moderate–high at low loading; reduced at higher concentrations due to ion toxicity	Can enhance short-term proliferation but inhibits long-term growth if overdosed	Limited or indirect differentiation effects; mainly antimicrobial function	ROS generation and Ag^+^ release cause oxidative stress; dose-dependent cytotoxicity
Zinc oxide nanoparticles (ZnO) [58]	Generally high at optimized concentrations; cytotoxic at high doses	Promotes proliferation of osteogenic and dermal cells at low wt%	Strong osteogenic differentiation (bone tissue engineering)	Zn^2+^ ion release enhances enzyme activity and signaling pathways
Silicates (nanosilicates/bioactive glass/Laponite) [58]	Very high viability due to bioactive and cytocompatible ion release	Strong stimulation of proliferation via ECM-like microenvironment	Highly effective osteogenic and chondrogenic differentiation	Ionic dissolution (Si, Mg, Li) activates osteogenic signaling and integrin binding
Carbon-based (Graphene, CNTs, CNDs) [59]	High viability at low concentration; CNTs may reduce viability if poorly dispersed	Graphene promotes proliferation; CNTs are variable depending on dispersion	Strong differentiation effects (especially neural and osteogenic lineages)	Electrical conductivity and surface interactions regulate focal adhesion and gene expression

**Table 6:** Physical properties of Nano ink formulation.

Nanomaterial System	Tensile/Compressive Strength	Elastic Modulus	Structural Stability	Mechanistic Basis of Reinforcement
Silver nanoparticles (AgNPs) [56,57]	Low–moderate improvement	Slight increase	Limited enhancement; requires polymer matrix support	Mainly particle dispersion reinforcement; weak interfacial bonding
Zinc oxide (ZnO NPs) [55,58]	Moderate improvement (up to ~20–40% increase in strength in hydrogels/composites)	Moderate increase	Improved but concentration-dependent stability	Ionic interactions (Zn^2+^) and particle–polymer crosslinking enhance stiffness
Silicates (nanosilicates/bioactive glass/Laponite) [50,51]	High improvement (often >2× strength in hydrogels)	High increase	Excellent structural fidelity and shape retention	Platelet-like structures form physical cross-linked networks → yield stress and shear-thinning behavior
Carbon-based (Graphene, CNTs, CNDs) [52,53,54]	Very high (graphene/CNTs show up to 2–10× reinforcement depending on loading)	Very high (especially CNTs)	High (graphene > CNTs due to dispersion issues)	Strong covalent π–π interactions + high aspect ratio reinforcement

## Data Availability

No new data were created or analyzed in this study.
